# Identifying Clinical Phenotypes in Moderate to Severe Acute Respiratory Distress Syndrome Related to COVID-19: The COVADIS Study

**DOI:** 10.3389/fmed.2021.632933

**Published:** 2021-03-11

**Authors:** Jean-Baptiste Lascarrou, Aurelie Gaultier, Thibaud Soumagne, Nicolas Serck, Bertrand Sauneuf, Michael Piagnerelli, Andre Ly, Francois Lejeune, Laurent Lefebvre, Sami Hraiech, Geoffrey Horlait, Julien Higny, Alain D'hondt, Stephane Gaudry, Romain Courcelle, Giuseppe Carbutti, Gauthier Blonz, Gregoire Ottavy, Nadia Aissaoui, Christophe Vinsonneau, Benoit Vandenbunder, Julien Textoris, Piotr Szychowiak, David Grimaldi, the COVADIS study group

**Affiliations:** ^1^Médecine Intensive Reanimation, CHU Nantes, Nantes Cedex, France; ^2^Plateforme de Méthodologie et Biostatistique, CHU Nantes, Nantes Cedex, France; ^3^Médecine Intensive Réanimation, CHU Besançon, Besançon, France; ^4^Unité de Soins Intensifs Clinique Saint Pierre, Ottignies, Belgium; ^5^Réanimation - Médecine Intensive, Centre Hospitalier Public du Cotentin, BP208, Cherbourg-en-Cotentin, France; ^6^Intensive Care, Centre Hospitalier Universitaire-Charleroi, Marie Curie, Université Libre de Bruxelles, Charleroi, Belgium; ^7^Service D'anesthésie-Réanimation Chirurgicale Unité de Réanimation Chirurgicale Polyvalente Hôpitaux Universitaires Henri Mondor, Créteil, France; ^8^Unité de Soins Intensifs Clinique Notre Dame de Grâce, Gosselies, Belgium; ^9^Réanimation Polyvalente Center Hospitalier du Pays d'Aix, Aix en Provence, France; ^10^Médecine Intensive Réanimation, Assistance Publique-Hôpitaux de Marseille, Hôpital Nord, Marseille, France; ^11^Centre d'Etudes et de Recherches sur les Services de Santé et Qualité de vie EA 3279, Aix- Faculté de médecine, Marseille Université, Marseille, France; ^12^Unité de Soins Intensifs, Centre Hospitalier Universitaire Dinant Godinne, Site Godinne, Yvoir, Belgium; ^13^Unité de Soins Intensifs, Centre Hospitalier Universitaire Dinant Godinne, Site Dinant, Dinant, Belgium; ^14^Unité de Soins Intensifs, Centre Hospitalier Universitaire Ambroise Paré, Mons, Belgium; ^15^Réanimation Médico-Chirurgicale CHU Avicennes, Université Sorbonne Paris Nord, Bobigny, France; ^16^Unité de Soins Intensifs, Centres Hospitaliers de Jolimont, La Louvière, Belgium; ^17^Unité de Soins Intensifs, Centre Hospitalier Regional Mons-Hainaut, Mons, Belgium; ^18^Médecine Intensive Réanimation, Center Hospitalier Départmental, Boulevard Stephane Moreau, La Roche Sur Yon, France; ^19^Médecine Intensive Réanimation, Hôpital Européen Georges Pompidou, Université de Paris, Paris Centre U 970 PARCC, Paris, France; ^20^Service de Médecine Intensive Réanimation Unité de Sevrage Ventilatoire et Réhabilitation Center Hospitalier de BETHUNE, Beuvry, France; ^21^Groupe des Anesthésistes Réanimateurs, Hôpital Privé d'Antony, Antony, France; ^22^Service de Réanimation, Hospices Civils de Lyon, Lyon, France; ^23^Laboratoire de Recherche bioMérieux-Hospices Civils de Lyon-Université de Lyon 1, Lyon, France; ^24^Médecine Intensive Reanimation, CHRU Tours, Tours, France; ^25^INSERM CIC 1415, CHRU Tours, Tours, France; ^26^CRICS-TriggerSEP Research Network, Tours, France; ^27^Soins Intensifs, Hôpital Erasme, Universite Libre Bruxelles, Bruxelles, Belgium

**Keywords:** COVID-19, ARDS, intubation, mechanical ventilation, phenotype

## Abstract

**Objectives:** Different phenotypes have been identified in acute respiratory distress syndrome (ARDS). Existence of several phenotypes in coronavirus disease (COVID-19) related acute respiratory distress syndrome is unknown. We sought to identify different phenotypes of patients with moderate to severe ARDS related to COVID-19.

**Methods:** We conducted an observational study of 416 COVID-19 patients with moderate to severe ARDS at 21 intensive care units in Belgium and France. The primary outcome was day-28 ventilatory free days. Secondary outcomes were mortality on day 28, acute kidney injury, acute cardiac injury, pulmonary embolism, and deep venous thrombosis. Multiple factor analysis and hierarchical classification on principal components were performed to distinguish different clinical phenotypes.

**Results:** We identified three different phenotypes in 150, 176, and 90 patients, respectively. Phenotype 3 was characterized by short evolution, severe hypoxemia, and old comorbid patients. Phenotype 1 was mainly characterized by the absence of comorbidities, relatively high compliance, and long duration of symptoms, whereas phenotype 2 was characterized female sex, and the presence of mild comorbidities such as uncomplicated diabetes or chronic hypertension. The compliance in phenotype 2 was lower than that in phenotype 1, with higher plateau and driving pressure. Phenotype 3 was associated with higher mortality compared to phenotypes 1 and 2.

**Conclusions:** In COVID-19 patients with moderate to severe ARDS, we identified three clinical phenotypes. One of these included older people with comorbidities who had a fulminant course of disease with poor prognosis. Requirement of different treatments and ventilatory strategies for each phenotype needs further investigation.

## Introduction

Coronavirus Disease (COVID-19) caused by severe acute respiratory syndrome coronavirus 2 (SARS-CoV-2) can have different clinical presentations, but respiratory symptoms predominate, and may induce acute respiratory distress syndrome (ARDS) (1).

On one hand, the clinical presentation of the respiratory disease is relatively homogenous. It mostly occurs among overweight men over 50 years old, with cardiovascular comorbidities and is characterized by severe hypoxemia and radiological ground glass opacities (2). On the other hand, some features of the disease are more heterogeneous: it can involve other organs such as the kidney (3) and the heart (4), other radiological patterns are described (5), and some ethnic specificities are observed. Regarding COVID-19 related ARDS, some experts advocate that patients can be separated into different sub-phenotypes (6, 7). In particular, experts hypothesized that COVID-19 patients with ARDS could be separated into two main phenotypes according to lung mechanical properties: some patients would have “early” ARDS (based on duration between symptoms onset and respiratory failure) with high compliance and low recruitability, whereas others patients would have “late” ARDS with low compliance and high recruitability. Those experts exert a physician to tailor respiratory therapy [such as tidal volume, positive end expiratory pressure (PEEP), or prone position session] for each phenotype individually. However, this theory has been challenged by others (8) who claim that identification of different phenotypes should be done using an unbiased approach in large cohorts of patients. Unsupervised classification methods have already caused the identification of several phenotypes in different intensive care unit (ICU) diseases, including ARDS (9). These strategies prevent cognitive biases (8), and simple bedside data could help better describe a previously unknown disease in an unbiased manner (10).

Indeed, most of the validated sub-phenotypes were based on biomarker dosages, which were time-consuming and somewhat costly. These caveats preclude sub-phenotyping of ARDS patients in routine critical care, while immediate interventions are often required. Conversely, phenotyping using simple clinical data could be immediately useful at bedside (9).

To investigate whether different clinical phenotypes of COVID-19 ARDS really coexist and lead to different outcomes, we performed a *post-hoc* analysis of patients included in the COVADIS study [i.e., patients with moderate or severe COVID-19 related ARDS admitted to 21 ICUs in Belgium and France (11–14)]. Patients were phenotyped according to two main determinants: demographic characteristics and respiratory characteristics upon initiation of mechanical ventilation. Classification was conducted without considering clinical outcomes, and we compared the outcomes of the different sub-phenotypes.

## Patients and Methods

### Study Design

This multicentric prospective observational study included 21 ICUs in France (*n* = 12) and Belgium (*n* = 9). The COVID-19 pandemic began in France in the 2nd week of March 2020 and 1 week later in Belgium. The inclusion period ended on April 15, 2020, with a 28-day follow-up.

### Patient Population

The inclusion criteria were as follows:

- Older than 18 years
- Moderate to severe ARDS according to the Berlin definition (15) (PaO_2_/FiO_2_ ratio <200 mmHg with a PEEP of at least 5 mmHg receiving invasive ventilation),
- Positive SARS-CoV-2 reverse transcriptase polymerase chain reaction (PCR).

The exclusion criteria were as follows:

- Cardiac arrest before ICU admission
- Extra corporeal membrane oxygenation (ECMO) requirement within the first 24 h of ICU admission.
- Chronic obstructive pulmonary disease with Global Initiative for Chronic Obstructive Lung Disease (GOLD) class 3 or 4 (16), or use of home oxygen.

### Data Collection

For this observational prospective multicenter study, all consecutive COVID-19 patients were screened in the participating centers. Patients fulfilling the inclusion and exclusion criteria were included in participating ICUs between March 10, 2020 and April 15, 2020. Each local investigator filled an eCRF to collect data (Castor EDC, Amsterdam, The Netherlands). We recorded demographic data, medical history, and comorbidities using the Charlson score (17), along with the history of chronic hypertension. We collected the PaO_2_/FiO_2_ ratio and the settings of the mechanical ventilator (MV) after intubation [tidal volume (Vt), PEEP, and plateau pressure]. We measured the duration of MV, administration of advanced therapies for acute respiratory failure (neuromuscular blocking agents, inhaled pulmonary vasodilators, prone-positioning, and ECMO), immunomodulatory agents (interleukin-6-receptor antagonists and corticosteroids), time from onset of symptoms and occurrence of acute kidney injury (AKI), acute cardiac injury (defined as a rise in troponin level over 10 times the normal threshold), the need for inotrope, pulmonary embolism (PE), and deep venous thrombosis.

### Primary Outcomes

The pre-specified primary endpoint was the number of ventilator-free days (VFD) at day 28 (18).

VFD at day 28 was determined as follow:

- VFDs = 0 if subject died within 28 days of mechanical ventilation,
- VFDs = 28 – *x* if the subject was successfully released from ventilation *x* days after initiation, and not reintubated until day 28.
- VFD = 0 if the subject was mechanically ventilated for >28 days.

The variable was dichotomized into “patients still ventilated or dead on day 28” (VFD = 0) vs. “patients weaned and alive on day 28” (VFD > 0).

### Secondary Outcomes

- Ventilator mode on day 14 according to four pre-defined categories: patient under volume/pressure assisted controlled or ECMO, pressure support mode, spontaneous breathing while extubated, and death.
- Acute cardiac injury was defined as a plasma troponin level > 10 times the upper normal range.
- Need for inotrope (dobutamine, epinephrine, milrinone, and/or levosimendan).
- AKI which was defined as a rise in serum creatinine of at least 50% as defined in Kidney Disease Improving Global Outcomes (KDIGO) stage 1 (19),
- Peak of creatinine.
- Need for renal replacement therapy (RRT).
- Deep venous thrombosis and PE.

### Ethics Approval

This study was approved by the appropriate regulatory committees in France (Commission National Informatique et Libertés n°2217488) and Belgium (Comité Ethique ERASME Université Libre de Bruxelles n°P2020/253) as per national regulations. Each patient was informed about the study. In the case of incompetency, next of kin was informed. The requirement for written informed consent was waived.

### Statistical Analysis

Continuous variables were described as median (25–75th percentiles) and categorical variables as number (percentage).

We performed a multiple factor analysis (MFA) with these variables followed by hierarchical clustering on principle components (HCPC) (20).

To perform the MFA, the quantitative variables were categorized according to commonly used cutoffs [body mass index (BMI), Charlson score, PaO_2_/FiO_2_ ratio], or according to the quartiles (age, duration between onset of symptoms and antiviral treatment, and compliance at baseline).

The variables were divided into two groups: demographic data (age, sex, BMI, and medical history) and respiratory data (PaO_2_/FiO_2_ ratio at baseline, compliance at baseline, co-infection, and duration between onset of symptoms and antiviral treatment). This was for balancing characteristics between past medical history and characteristics (especially respiratory characteristics) of the disease. Regarding comorbidities, we gathered them based on common pathophysiology: chronic hypertension / diabetes mellitus without complication / chronic respiratory failure / history of gastroduodenal ulcer / history of cancer / connectivitis or HIV / mild to moderate hepatic failure / dementia, hemiplegia or history of stroke / moderate chronic kidney, diabetes mellitus with complication / congestive heart failure, and ischemic cardiomyopathy.

Finally, regarding the respiratory disease, we included delay of symptoms, PaO2/FiO2 ratio, and static compliance of the respiratory system calculated as Crs = (Plateau pressure – PEEP)/ Vt and presence of a co-infection at baseline.

MFA, which belongs to a family of descriptive methods, is an extension of correspondence analysis that assesses contingency tables exploring simultaneous relationships among variables structured in groups to describe correlations between variables and patients. It appears to be a counterpart of principal component analysis for categorical data, used to detect and represent underlying structures in a dataset as points in a low-dimensional space (21).

We subjected the MFA results to HCPC using Ward's method to merge similar patients into clusters. HCPC is one of the leading data descriptive methods. It is used to group individuals with similar patterns of responses from quantitative data. The objective is to classify individuals into groups that are as homogeneous as possible (22). HCPC has been evaluated with higher stability than latent class analysis (LCA) in previous literature (23) without assumptions on the existence of latent variables. The optimal number of clusters was determined from the dendrogram, inertia criterion, and clinical relevance. On the dendrogram, significant changes between two levels of cuts suggest an optimal number of groups (21). For the inertia criterion (24), we defined the number (N) of clusters as the number after which the increase of between-cluster inertia from N-1 to N clusters was more important than the inertia's increase from N to N+1 clusters. To do this for each N, we calculated the ratio between the value of the increase in between-cluster inertia from N-1 to N clusters, divided by the increase in between-cluster interest from N to N+1 clusters (N ranging from the number of patients to 1). We selected the number of clusters as N with minimal ratio.

To visualize the clusters, a plot was produced by projecting the patients and center of gravity of each cluster, using the first two principal components.

Classification was conducted without consideration of clinical outcomes. The clusters thus identified were described by comparing the frequencies of different variables using the Chi-square test or Fisher's test, depending on the number of patients, for categorical variables, and analysis of variance (ANOVA) or the Kruskal–Wallis test, if the normality tested by a Shapiro Wilks test, has not been concluded for quantitative variables. Two close phenotypes were compared using correction of the alpha risk by the Holm method. R 3.6.0 was used for statistical analyses. *P* < 0.05 was considered statistically significant.

## Results

### Baseline Characteristics

**Figure 1:**
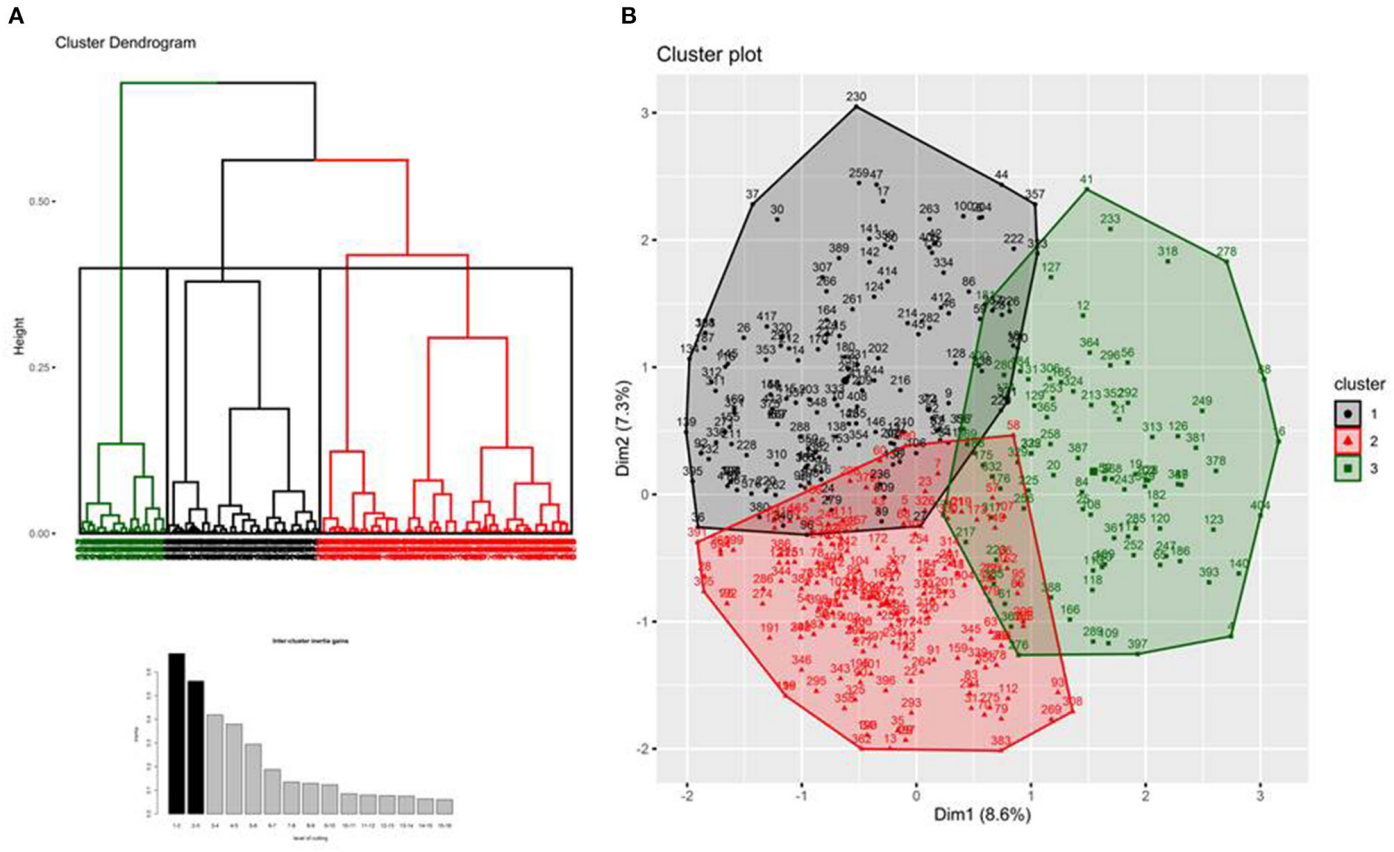
Hierarchical clustering tree (panel **A**), bar plot of inertia gains (panel **A**), and factor map of candidates' characteristics (panel **B**).

A total of 417 patients were included in the study, and one patient withdrew consent. By analyzing the baseline characteristics of the 416 remaining patients (demographic data, comorbidities, and COVID-19 related variables) with multiple component analysis independent of clinical outcomes, we observed that three different phenotypes could be identified (Figure 1 and Supplementary Figure 1). The discriminating variables that allow separating these three phenotypes are shown in the Supplementary Material. Overall, comorbidities, duration of symptoms, and compliance with the respiratory system were the most discriminating variables among patients, while age and BMI were weakly discriminating in this analysis (Supplementary Material).

As shown in Figure 1A, phenotype 3 (*N* = 90) was first separated from the two others. It was characterized by old age, presence of severe comorbidities (at least two points in the Charlson comorbidity index), short symptom duration, and severe hypoxemia. Phenotypes 1 and 2 were closer to each other. Phenotype 1 (*N* = 176) was mainly characterized by the absence of comorbidities, relatively high compliance, and a long duration of symptoms, whereas phenotype 2 (*N* = 150) was characterized by female sex, and presence of mild comorbidities such as uncomplicated diabetes or chronic hypertension. The compliance in phenotype 2 was lower than that in phenotype 1, with higher plateau and driving pressure. Conversely, the PaO_2_/FiO_2_ ratio was similar (Table 1). Patients of all the three phenotypes were treated similarly with low Vt, high PEEP, and frequent use of prone positioning, irrespective of their phenotype (Table 1).

**Table 1:** Patients characteristics according to phenotype.

**Characteristics *N* = 416**	**Total *N* = 416**	**Phenotype 1 *N* = 150**	**Phenotype 2 *N* = 176**	**Phenotype 3 *N* = 90**	***P-*value^a^**	**Adjusted *p-*value^b^ (1 vs. 2)**
**Main clinical characteristics analyzed for phenotyping**						
Age, median (IQR)anitha *N* = 416	63 (55–71)	63 (58–70)	60 (52–68)	71 (64–73.0)	<0.001	0.004
Sex, male, *n* (%)	321 (77.2)	129 (86)	119 (67.6)	73 (81.1)	<0.001	<0.001
Body mass index, median (IQR)anitha *N* = 399	29.05 (26.1–32.7)	28.1 (25.8–30.0)	29.7 (26.1–33.3)	30.9 (27.5–34.9)	<0.001	0.004
History of chronic hypertension, *n* (%)	235 (56.5)	65 (43.3)	99 (56.2)	71 (78.9)	<0.001	<0.001
Diabetes mellitus, *n* (%)	78 (18.75)	11 (7.3)	51 (29)	16 (17.8)	<0.001	<0.001
CKD, *n* (%)	33 (7.9)	12 (8)	2 (1.1)	19 (21.1)	<0.001	<0.001
Myocardial infarction or chronic cardiac failure, *n* (%)	49 (11.8)	12 (8)	5 (2.8)	32 (35.6)	<0.001	<0.001
Charlson score, median (IQR)anitha *N* = 416	1 (0–2)	0.5 (0–2)	0 (0–1)	3 (2–4)	<0.001	0.008
**Disease characteristics included in phenotyping**						
Time from symptoms onset (days), median (IQR) *N* = 355	8 (5–10)	10 (9–12)	7 (6–8)	4 (3.0–5.5)	<0.001	<0.001
Co-infection, *n* (%)	48 (11.5)	29 (19.3)	8 (4.6)	11 (12.2)	<0.001	<0.001
FiO2 (%), median (IQR)	80 (60–100)	75 (60–100)	80 (60–100)	85 (60–100)	0.044	0.17
P/F ratio (mmHg),anitha median (IQR) *N* = 413	124 (88–158)	130 (107–151)	124 (87–165.5)	96 (75–159)	0.022	0.773
Compliance rs, (mL/cm H_2_O) median (IQR), *N* = 366	35.4 (28.7–44.9)	43 (36.7–50)	31.1 (26.7–37.2)	33.3 (26.6–42.8)	<0.001	<0.001
**Other characteristics not included in phenotyping**						
Country France (vs. Belgium)	240 (57.7)	90 (60)	99 (56.3)	51 (56.7)	0.77	>0.99
Tidal volume, (mL/kg of IBW), median (IQR) *N* = 397	6.1 (5.8–6.6)	6.1 (5.8–6.5)	6.1 (5.8–6.6)	6.1 (5.8–6.9)	0.98	>0.99
Total PEEP (cm H_2_O), median (IQR) *N* = 415	12 (10–14)	12 (10–14)	12 (10–14)	10 (10–13)	0.11	0.74
Plateau pressure (cm H_2_O), median (IQR) *N* = 366	23 (21–26)	22 (20–24)	24 (22–28)	23.5 (21–27)	<0.001	<0.001
Driving pressure (cm H_2_O), median (IQR) *N* = 366	12 (9–14)	10 (8–12)	12.5 (11–15)	12 (10–15)	<0.001	<0.001
Neuromuscular blockade, *n* (%)	350 (84.1)	124 (82.7)	151 (85.8)	75 (83.3)	0.72	>0.99
Inhaled nitric oxide, *n* (%)	48 (11.5)	14 (9.3)	23 (13.1)	11 (12.2)	0.56	>0.99
Prone position, *n* (%)	330 (79.3)	121 (80.7)	144 (81.8)	65 (72.2)	0.17	0.33
Corticosteroids, *n* (%)anitha *N* = 394^c^	85 (21.6)	28 (19.7)	40 (24)	17 (20)	0.62	0.90
Inhibitor of IL-6, *n* (%)	10 (2.4)	1 (0.7)	7 (4)	2 (2.2)	0.17	0.33

*IQR, Inter-quartile range; IBW, ideal body weight; P/F ratio, PaO_2_/FiO_2_ ratio; CKD, chronic kidney disease; Compliance rs, compliance of respiratory system; PEEP, Postive end expiratory pressure; IL, Interleukin*.

a *P-value from Kruskal-Wallis, or Chi-square test*.

b *Adjusted P-value from the comparison of phenotypes 1 and 2 (Mann-Whitney Wilcoxon or Fisher test) corrected by the Holm method*.

c *Some patients were included in a double-blind RCT of steroids vs. placebo (NCT02517489) and were considered as missing data*.

### Primary Outcomes

**Figure 2:**
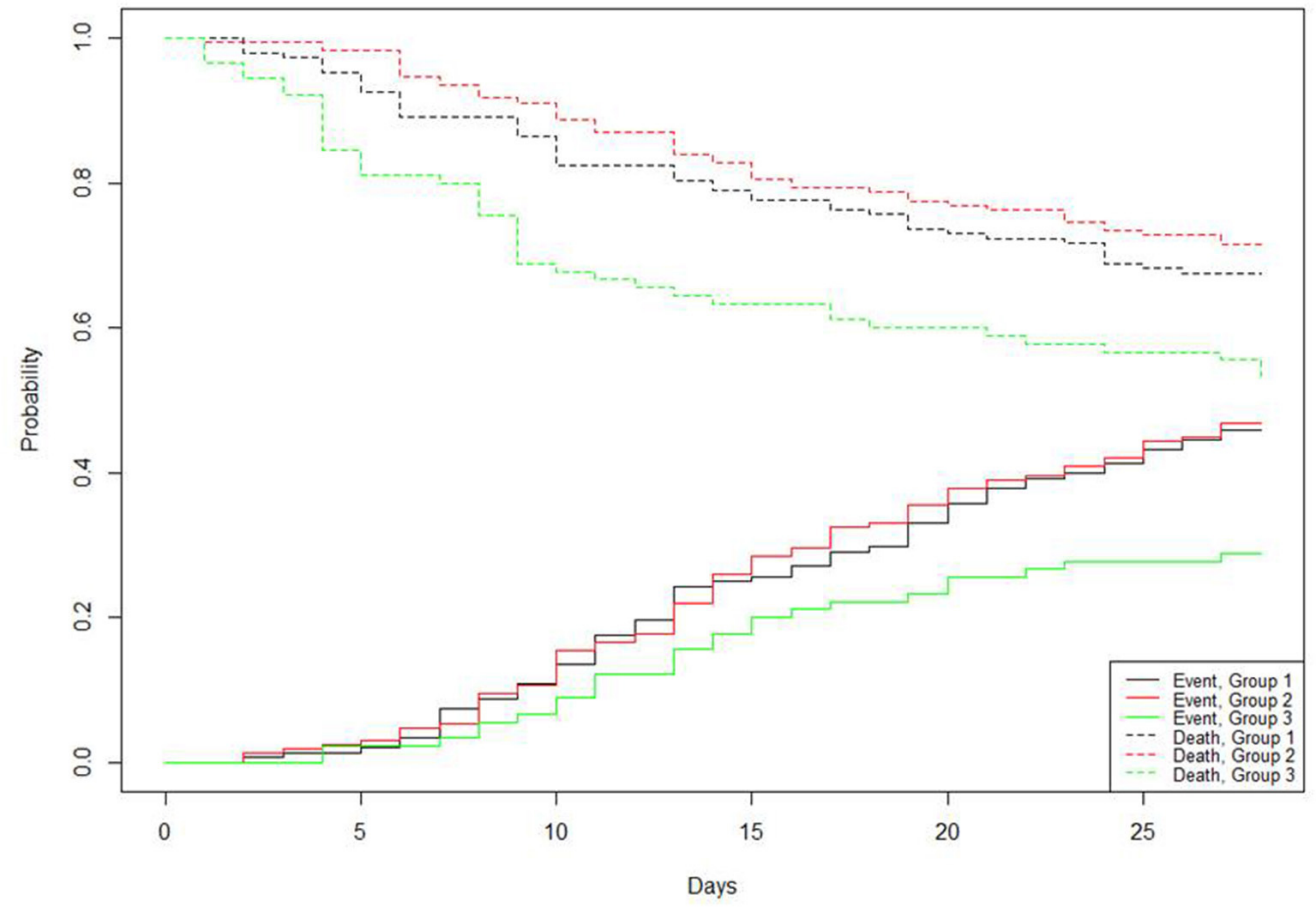
Probability of dying or being weaned over time during 28 days.

A total of 407 patients were available on day 28 for follow-up. As shown in Table 2, patients classified into phenotype 3 had lower number of VFDs on day 28. The probability of death was high in this phenotype, whereas the probability of breathing without assistance was low (Figure 2). Conversely, phenotypes 1 and 2 had similar numbers of VFDs and survival rates (Table 2 and Figure 2).

**Table 2:** Outcome according to phenotype.

**Characteristics**	**Total *N* = 416**	**Phenotype 1 *N* = 150**	**Phenotype 2 *N* = 176**	**Phenotype 3 *N* = 90**	***P-*value*^a^***	**Adjusted *p*-value^b^ (1 vs. 2)**
**Ventilatory mode at day 14**, ***n*** **(%)**						
- Death - Controlled mode or under VV-ECMO - Pressure Support - Extubated	93 (22.5) anitha 140 (34.0) anitha87 (21.1) anitha92 (22.3)	31 (20.8) anitha52 (34.9) anitha31 (20.8) anitha35 (23.5)	29 (16.8) anitha59 (34.1) anitha43 (24.9) anitha42 (24.3)	33 (36.7) anitha29 (32.2) anitha13 (14.4) anitha15 (16.7)	0.02	> 0.99
Needs for ECMO, *n* (%)	49 (11.8)	14 (9.3)	30 (17.1)	5 (5.6)	0.01	0.02
Alive at day 28, *n* (%)	273 (66.4)	101 (67.8)	124 (72.1)	48 (53.3)	0.009	0.95
VFD on day 28, median (IQR)anitha *N* = 407	0 (0–13)	0 (0–13)	0 (0–14)	0 (0–8)	0.03	>0.99
Breathing without assistance at day 28, *n* (%)	173 (42.5)	68 (45.95)	79 (46.75)	26 (28.89)	0.01	>0.99
Acute kidney injury, *n* (%)	225 (55.1)	82 (55.41)	81 (47.65)	62 (68.89)	0.005	0.41
Need for RRT, *n* (%)	78 (19.1)	32 (21.33)	27 (15.34)	19 (21.11)	0.31	0.42
Peak of creatinine, median (IQR)anitha *N* = 401	126 (84–280)	123 (86–300)	106 (75–232)	183 (108–390)	<0.001	0.14
Acute cardiac injury, *n* (%)	31 (7.5)	5 (3.33)	14 (7.95)	12 (13.33)	0.016	0.25
Need for inotropes, *n* (%)	30 (7.2)	11 (7.33)	8 (4.55)	11 (12.22)	0.07	0.81
Pulmonary embolism, *n* (%)	59 (14.4)	30 (20.3)	17 (9.9)	12 (13.3)	0.03	0.03
Deep veinous thrombosis, *n* (%)	39 (9.6)	14 (9.5)	19 (11.1)	6 (6.7)	0.51	>0.99

*VV-ECMO, venovenous extracorporeal membrane oxygenation; VFD, ventilator-free days; IQR, Inter-quartile range; RRT, Renal replacement therapy*.

a *P-value from Kruskal-Wallis, or Chi-square test*.

b *Adjusted P-value from the comparison of phenotypes 1 and 2 (Mann-Whitney Wilcoxon or Fisher test) corrected by the Holm method*.

### Secondary Outcomes

Regarding key pre-specified secondary outcomes, we observed that phenotype 3 was frequently associated with the need for inotrope for cardiac failure (Table 2). Although phenotype 3 was also frequently associated with AKI, the rate of renal replacement therapy did not differ across phenotypes. The outcome between phenotypes 1 and 2 differed for ECMO implantation being more frequent in phenotype 2 (17 vs. 9%; *P* = 0.02), whereas pulmonary embolism was more frequent in phenotype 1 (20 vs. 10%; *P* = 0.03). However, the occurrence of deep venous thrombosis was similar (Table 2).

## Discussion

In this observational study of moderate to severe ARDS complicating COVID-19 in France and Belgium, we attempted to identify different clinical phenotypes of this new disease using simple bedside available clinical data. Using a multiple factor analysis, we identified three main clinical sub-phenotypes that had different clinical characteristics, and among them, one had the worst outcome.

Phenotypes have been identified in the ICU in heterogeneous syndromes such as ARDS or sepsis (25). Phenotyping may be used for prognostic enrichment (i.e., identifying a subset of patients with a high likelihood of a given outcome) or for studying how treatment effects vary across sub-phenotypes (predictive enrichment) (26, 27). Phenotyping may also allow a better understanding of these syndromes' complexities and identifying more homogeneous groups of patients. The sub-phenotypes in these studies were mainly based on biomarker dosages (26) or transcriptomic studies (28), which may be difficult to translate into clinical phenotypes in routine practice (9). Phenotyping has also been used in specific diseases such as asthma (29), post-resuscitation shock, or leptospirosis (30). Indeed, in infectious diseases, the determinant of host-pathogen interaction can lead to different phenotypes in terms of severity or clinical symptoms (31). Phenotyping may be useful in this setting for identifying a subset of patients with a high likelihood of a given outcome and to better describe a previously unknown disease in an unbiased manner.

In this study, we identified three main phenotypes in COVID-19 patients with moderate to severe ARDS. The most specific phenotype (phenotype 3) was less frequent (21% of the cohort) and prevalent among old and comorbid patients. Therefore, its association with worse outcomes was not surprising. Nevertheless, this result highlights the importance of including previous clinical conditions in phenotyping studies. In our view, the most interesting results regarding phenotype 3 are that it includes patients with the lowest duration of symptoms, poor hypoxemia, and low compliance, and that these patients had high AKI occurence, required frequent inotrope, and ultimately high probability of death. Thus, we hypothesize that these patients suffered from a fulminant form of COVID-19 with rapid and massive lung injury and early systemic spread. RRT rate, a more patient-centered outcome, was similar across the phenotypes, suggesting that other factors may be involved (13, 32). In addition to this striking and specific phenotype, we identified two closer phenotypes (phenotype 1 and 2) with less differences in terms of clinical characteristics. Phenotype 1 had the longest duration of symptoms and the highest compliance, whereas phenotype 2 included predominantly females and patients with minor comorbidities who had lower compliance and shorter durations of symptoms. Interestingly, we did not find a relationship between low compliance with long duration of symptoms, as hypothesized by some authors. The absence of a relationship between duration of symptoms and compliance has already been observed in a monocentric study (33), while another study did not show any relationship between compliance and thoracic computed tomography-scan (34) questioning the hypothetic model of high and low compliance phenotypes.

Lastly, as day-28 mortality and duration of ventilation were strictly similar between these two sub-phenotypes, one may question their clinical relevance (35). It should be noted that despite similar day-28 survival, the rates of ECMO implantation and pulmonary embolism differed between these two phenotypes, possibly due to more alveolar injury in phenotype 2 and more vascular injury in phenotype 1 (36); thus highlighting the possible existence of hypo- and hyper-inflammatory phenotypes in ARDS related to COVID-19. These results may be considered with caution, as no standard procedures were defined for ECMO implantation or for prevention and detection of PE (11). As different treatments are now available for COVID-19 with conflicting results according to severity of patients, the different responses to corticosteroids (37) and/or remdesivir (38) during study inclusion and subgroup analysis can be tested further.

Our study has several strengths. It considered one of the largest multicentric cohorts of COVID-19 patients with well-defined ARDS. This cohort is in line with previous findings regarding COVID-19 related ARDS in other countries (39, 40). Patients were mostly overweight males, aged between 50 and 70 years, with mild cardiovascular comorbidities. Although each center has separate management protocols for ventilator support, we observed it in line with ARDS guidelines, (41) physicians set Vt near 6 mL/kg of ideal body weight, PEEP at moderate–high level, used largely prone positioning, and paralysis, reinforcing the relationship between phenotype and outcome. We considered comorbidities in our phenotyping study, highlighting their role in the pathophysiology of COVID-19 related ARDS. Interestingly, the distribution of each phenotype in the two participating countries (France and Belgium) was nearly identical, which was consistent with the center effect. Finally, other researchers have recently found three distinct phenotypes using their own datasets and different methods of grouping patients, but including patients outside the ICU (42).

Our study has several limitations. Interventions were not randomized, so we could not study how treatment effects vary across phenotypes, an approach named predictive enrichment (26, 27). Due to paucity of time during the COVID-19 crisis, we limited the number of collected variables and we were unable to report important data such as the use of angiotensin-converting enzyme inhibitors, focal or non-focal lung morphology, or inflammatory markers. Additionally, we did not report daily ventilator settings but only the settings after intubation; however, it seems that ARDS phenotypes remain identifiable during the initial days (43). We did not collect severity scores, but these scores were used to compare patients with different diseases in the ICU, and Charlson score, associated with sex and age, has been shown to predict mortality with good accuracy (44). Lastly, recent literature highlights significant difference between patients hospitalized during first and second wave (45, 46) and our analyze is based only on first wave patients. Unfortunately, we were not able to validate our findings in an external cohort especially including patients from both waves but prepare a dedicated file to help clinicians who share with us interest in this project.

## Conclusion

In COVID-19 patients with moderate to severe ARDS, we identified three clinical phenotypes based on patient and disease characteristics. One of these included old people with comorbidities who had a fulminant course of disease with poor prognosis. Despite differences in the compliance of the respiratory system on other days, the 28-day outcome was similar. Our study allows the early identification of clinical phenotypes. The requirement of different treatment and ventilatory strategies for each phenotype needs further investigation.

## Data Availability Statement

The data analyzed in this study is subject to the following licenses/restrictions: data sharing on request to corresponding author after Ethics Committe approval. Requests to access these datasets should be directed to Jean-Baptiste Lascarrou, jeanbaptiste.lascarrou@chu-nantes.

## Ethics Statement

The studies involving human participants were reviewed and approved by Commission Nationale Informatique et Libertés n°2217488. Written informed consent for participation was not required for this study in accordance with the national legislation and the institutional requirements.

## COVADIS Study Group Investigators

- Nadia Aissoui, Médecine Intensive Réanimation, European University Hospital, Paris, France
- Patrick Biston, intensive care CHU-Charleroi, Marie Curie. Université Libre de Bruxelles 140, Chaussée de Bruxelles. 6042-Charleroi, Belgium
- Gauthier Blonz, Medecine Intensive Reanimation, CHD Vendée, site de la Roche sur Yon, Les Oudairies, 85000 La Roche Sur Yon, France
- Gwenhael Colin, Medecine Intensive Reanimation, CHD Vendée, site de la Roche sur Yon, Les Oudairies, 85000 La Roche Sur Yon, France
- Romain Courcelle, Department of Intensive Care, Center Hospitaliers de Jolimont, La Louvière, Belgium
- Alain D'Hondt, Unités de soins intensifs CHU Ambroise Paré, Mons, Belgium
- Oriane de Maere, Department of Intensive Care,CHR Mons-Hainaut, Mons, Belgium
- Nathan Ebstein, Réanimation médico-chirurgicale CHU Avicennes, Université Sorbonne Paris Nord, Bobigny, France
- Stephan Ehrmann, Médecine Intensive Réanimation, CHRU Tours, Tours, France
- Frederic Foret, Unité de soins intensifs, CHU Dinant Godinne, site Dinant, Belgium
- Lionel Haentjens, Unités de sois intensifs CHU Ambroise Paré, Mons, Belgium
- Thibault Helbert, Réanimation polyvalente Center Hospitalier du pays d'Aix, Aix en Provence, France
- Julien Higny, Unité de soins intensifs, CHU Dinant Godinne, site Dinant, Belgium
- Geoffroy Horlait, CHU UCL Namur, Site Godinne, Av. Dr. G. Therasse 1 5530, Yvoir, Belgium
- Sami Hraiech, Médecine Intensive Réanimation, Assistance Publique-Hôpitaux de Marseille, Hôpital Nord, 13015, Marseille, France
- Stéphane Gaudry, Réanimation médico-chirurgicale CHU Avicennes, Université Sorbonne Paris Nord, Bobigny, France
- Laurent Lefebvre, Réanimation polyvalente Center Hospitalier du pays d'Aix, Aix en Provence, France
- André Ly, Service d'anesthésie-réanimation chirurgicale Unité de réanimation chirurgicale polyvalente Hôpitaux Universitaires Henri Mondor, Créteil, France
- Jean-Baptiste Mesland, Department of Intensive Care, Center Hospitaliers de Jolimont, La Louvière, Belgium
- Celine Monard, Service de réanimation, Hospices Civils de Lyon, 5 Place D'Arsonval, Lyon, France
- Nicolas Mongardon, Service d'anesthésie-réanimation chirurgicale Unité de réanimation chirurgicale polyvalente Hôpitaux Universitaires Henri Mondor, Créteil, France
- Grégoire Ottavy, Médecine Intensive Réanimation, CHU Nantes, 30 Bd Jean Monnet, 44000 Nantes, France
- Thomas Pasau, CHU UCL Namur, Site Godinne, Av. Dr. G. Therasse 1 5530, Yvoir, Belgium
- Michael Piagnerelli, Intensive Care. CHU-Charleroi, Marie Curie. Université Libre de Bruxelles 140, Chaussée de Bruxelles. 6042-Charleroi, Belgium
- Gael Piton, Médecine Intensive Réanimation, CHU Besançon, 3 Boulevard FLEMING, 25030 Besançon, France
- Ester Ponzetto, Unité de soins intensifs, Clinique Saint Pierre, Ottignies, Belgium
- Bertrand Sauneuf, Réanimation - Médecine Intensive, Centre Hospitalier Public du Cotentin, BP208, 50102 Cherbourg-en-Cotentin, France
- Piotr Szychowiak, Szychowiak, Médecine Intensive Réanimation, CHRU Tours, Tours, France
- Nicolas Serck, Unité de soins intensifs, Clinique Saint Pierre, Ottignies, Belgium
- Caroline Sejourne, Service de Médecine Intensive Réanimation, CH Germon et Gauthier, Béthune, France
- Morgane Snacken, Soins Intensifs, Hôpital Erasme, ULB, Route de Lennik 808, 1070 Bruxelles, Belgium
- Thibaut Soumagne, Médecine Intensive Réanimation, CHU Besançon, 3 Boulevard FLEMING, 25030 Besançon, France
- Xavier Souloy, Réanimation - Médecine Intensive, Centre Hospitalier Public du Cotentin, BP208, 50102 Cherbourg-en-Cotentin, France
- Aude Sylvestre, Médecine Intensive Réanimation, Assistance Publique-Hôpitaux de Marseille, Hôpital Nord, 13015, Marseille, France
- Nicolas Tartrat, Groupe des anesthésistes réanimateurs, Hôpital Privé d'Antony, Antony, France
- Cedric Vanbrussel, Unité de soins intensifs, Clinique Notre Dame de Grâce, Gosselies, Belgium
- Benoit Vandenbunder, Groupe des anesthésistes réanimateurs, Hôpital Privé d'Antony, Antony, France
- Christophe Vinsonneau, Service de Médecine Intensive Réanimation, CH Germon et Gauthier, Béthune, France.

## Author Contributions

J-BL and DG were responsible for the study concept and design. J-BL, AG, and DG: analysis and interpretation of the data and drafting of the manuscript. All authors: acquisition of data, critical revision of the manuscript for important intellectual content, and read and approved the final manuscript. The corresponding author had full access to all the data in the study and final responsibility for the decision to submit for publication.

## Conflict of Interest

JT is a part-time employee of bioMérieux, an IVD company, and Hospices Civils de Lyon, a university hospital. The remaining authors declare that the research was conducted in the absence of any commercial or financial relationships that could be construed as a potential conflict of interest.
