# Supplementary material for: Identifying Clinical Phenotypes in Moderate to Severe Acute Respiratory Distress Syndrome Related to COVID-19: The COVADIS Study
Source: Front Med (Lausanne). 2021 Mar 11;8:632933. doi: 10.3389/fmed.2021.632933 (PMC7991403; doi:10.3389/fmed.2021.632933)
Supplement: Supplementary file 1 [file Data_Sheet_1.DOCX]

Electronic Supplementary Appendix

Identification of clinical sub-phenotypes

in moderate to severe acute respiratory distress syndrome related to COVID-19

The COVADIS multi-center observational study.

By Lascarrou *et al*.

Supplementary figure 1


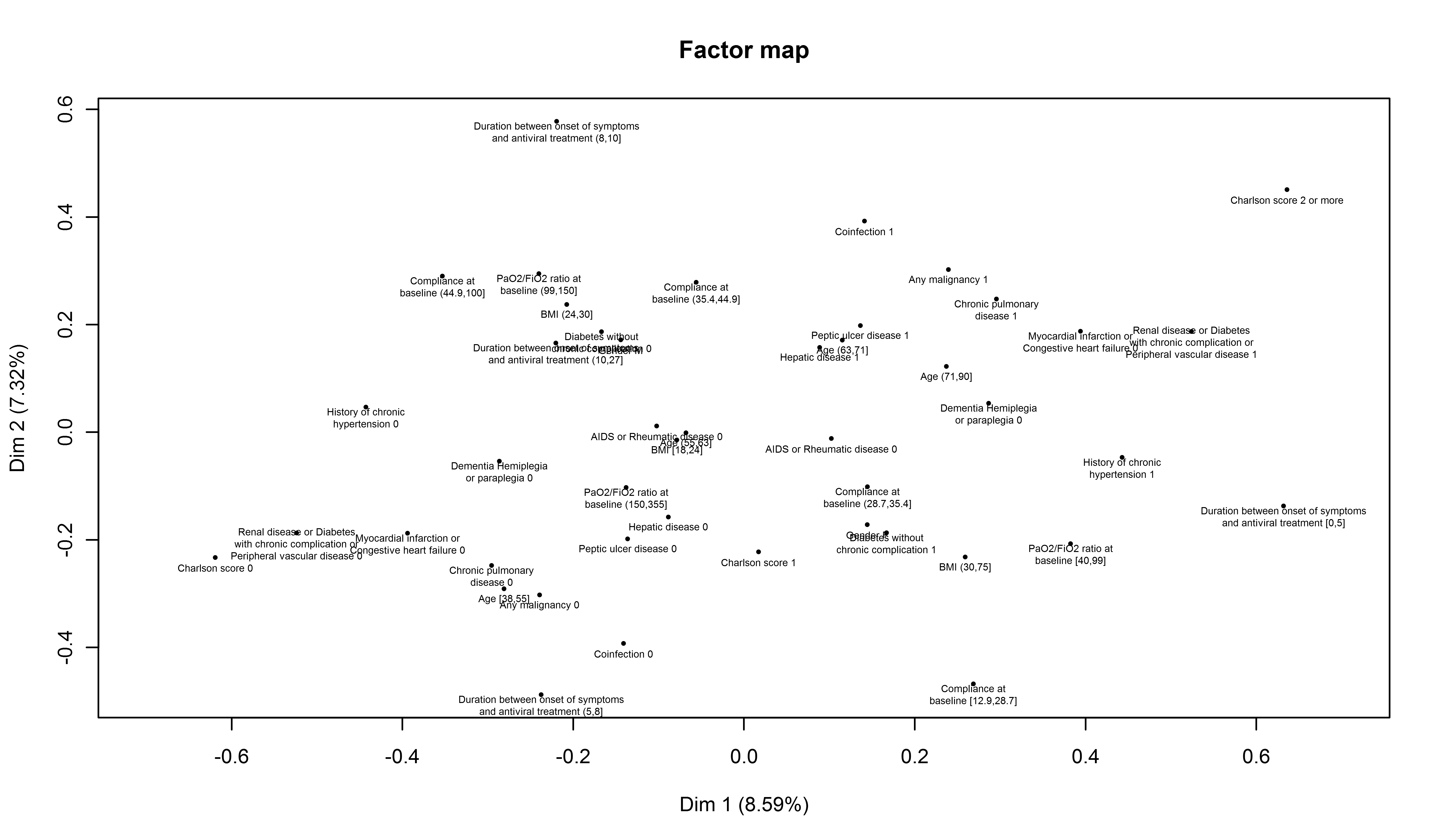


| **Phenotype 1 (N = 165)** | **Cla/Mod** | **Mod/Cla** | **Global** | **v.test** | **p.value** |
| --- | --- | --- | --- | --- | --- |
| Absence of chronic hypertension | 61.9 | 67.9 | 43.5 | 8.2 | < 0.001 |
| Compliance rs: 4^th^ quartile (44.9, 100) (mL/cm H_2_O) | 70.3 | 47.3 | 26.7 | 7.6 | < 0.001 |
| Symptoms duration: 4^th^ quartile (10, 27) days | 79.4 | 35.1 | 17.5 | 7.6 | < 0.001 |
| Symptoms duration: 3^rd^ quartile (8, 10) days | 76.2 | 38.8 | 20.2 | 7.6 | < 0.001 |
| Charlson Comorbidity index = 0 | 56.6 | 60 | 42.1 | 6 | < 0.001 |
| Absence of diabetes mellitus | 45.9 | 93.9 | 81.3 | 5.7 | < 0.001 |
| Compliance rs : 3^rd^ quartile (35.4, 44.9) (mL/cm H_2_O) | 62 | 37.6 | 24 | 5.2 | < 0.001 |
| Male gender | 46.1 | 89.7 | 77.2 | 5.1 | < 0.001 |
| Absence of CKD or PAD or complicated diabetes | 44.5 | 95.1 | 84.9 | 5 | < 0.001 |
| Overweight patients (BMI: 24–30 kg/m^2^) | 50.7 | 61.8 | 48.3 | 4.5 | < 0.001 |
| Absence of myocardial infarction and chronic cardiac failure | 42.5 | 94.5 | 88.2 | 3.3 | < 0.001 |
| Presence of Co-infection | 56.2 | 16.4 | 11.5 | 2.4 | 0.015 |
| Age: 4th quartile (71, 90) years | 30.7 | 18.8 | 24.3 | -2.1 | 0.034 |
| Absence of co-infection | 37.5 | 83.6 | 88.5 | -2.4 | 0.015 |
| Charlson Comorbidity index = 1 | 27.6 | 17.6 | 25.2 | -2.9 | 0.003 |
| PaO_2_/FiO_2_ <100 mmHg | 28.7 | 23.6 | 32.7 | -3.2 | 0.001 |
| Previous myocardial infarction or chronic cardiac failure | 18.4 | 5.4 | 11.8 | -3.3 | < 0.001 |
| Charlson Comorbidity index = 2 | 27.2 | 22.4 | 32.7 | -3.6 | < 0.001 |
| Obese patients (BMI >30 Kg/m^2^) | 27.3 | 28.5 | 41.3 | -4.3 | < 0.001 |
| Presence of CKD or PAD or complicated diabetes | 12.7 | 4.8 | 15.1 | -5.0 | < 0.001 |
| Female gender | 17.9 | 10.3 | 22.8 | -5.1 | < 0.001 |
| Diabetes mellitus | 12.8 | 6.1 | 18.7 | -5.7 | < 0.001 |
| Symptoms duration: 2^nd^ quartile (5, 8) days | 21.1 | 18.2 | 34.1 | -5.7 | < 0.001 |
| Compliance rs: 2^nd^ quartile (28.7, 35.4) (mL/cm H_2_O) | 14 | 8.5 | 24 | -6.3 | < 0.001 |
| Compliance rs: 1^st^ quartile (12.9, 28.7) (mL/cm H_2_O) | 10.5 | 6.7 | 25.2 | -7.5 | < 0.001 |
| Symptoms duration: 2^nd^ quartile (0, 5) days | 11.1 | 7.9 | 28.1 | -7.8 | < 0.001 |
| Chronic hypertension | 22.5 | 32.1 | 56.5 | -8.2 | < 0.001 |

ESM Table 1: Variables associated with phenotype 1

**+**

**+**

Variables positively

associated with the phenotype

Variables negatively

associated with the phenotype

Cla/Mod: Phenotype frequency in the variable modality
Mod/Cla: Variable modality frequency in the phenotype
Global: Variable modality overall frequency

v. test and p value are obtained by performing a test corresponding to the hypergeometric distribution and calculating the probability of observing a more extreme value than that observed under the assumption of a draw with discount.

Compliance rs: Compliance with the respiratory system

CKD: chronic kidney disease, BMI: Body mass index

PAD: Peripheral arterial disease.

ESM Table 2. Variables associated with phenotype 2

| **Phenotype 2 (N = 142)** | **Cla/Mod** | **Mod/Cla** | **Global** | **v.test** | **p.value** |
| --- | --- | --- | --- | --- | --- |
| Symptoms duration: 2^nd^ quartile (5.8) days | 73.2 | 73.2 | 34.1 | 12.1 | < 0.001 |
| Compliance rs: 2^nd^ quartile (28.7, 35.4) (mL/cm H_2_O) | 64 | 45.1 | 24 | 7 | < 0.001 |
| Diabetes mellitus | 66.7 | 36.6 | 18.7 | 6.5 | < 0.001 |
| Charlson Comorbidity index =1 | 58.1 | 43 | 25.2 | 5.8 | < 0.001 |
| Male gender | 58.9 | 39.4 | 22.8 | 5.6 | < 0.001 |
| Absence of CKD or PAD or complicated diabetes | 38 | 94.4 | 84.9 | 4.1 | < 0.001 |
| Chronic hypertension | 41.7 | 69 | 56.5 | 3.7 | < 0.001 |
| Compliance rs: 1^st^ quartile (12.9,28.7) (mL/cm H_2_O) | 47.6 | 35.2 | 25.2 | 3.3 | 0.001 |
| Absence of neurological comorbidity* | 35.8 | 98.6 | 94 | 3 | 0.003 |
| Absence of myocardial infarction and chronic cardiac failure | 36.5 | 94.4 | 88.2 | 2.9 | 0.004 |
| Obese patients (BMI >30 Kg/m^2^) | 41.9 | 50.7 | 41.4 | 2.8 | 0.006 |
| Absence of cancer | 36.2 | 94.4 | 88.9 | 2.6 | 0.009 |
| PaO_2_/FiO_2_: 100–150 mmHg | 41.6 | 47.2 | 38.7 | 2.5 | 0.011 |
| Absence of chronic pulmonary disease | 36.4 | 91.6 | 85.8 | 2.5 | 0.014 |
| Absence of co-infection | 36.1 | 93.7 | 88.5 | 2.4 | 0.015 |
| PaO_2_/FiO_2_: <100 mmHg | 26.5 | 25.3 | 32.7 | -2.3 | 0.021 |
| Co-infection | 18.7 | 6.3 | 11.5 | -2.4 | 0.015 |
| Chronic pulmonary disease | 20.3 | 8.5 | 14.2 | -2.5 | 0.014 |
| Cancer | 17.4 | 5.6 | 11.1 | -2.6 | 0.009 |
| Previous myocardial infarction or chronic cardiac failure | 16.3 | 5.6 | 11.8 | -2.9 | 0.004 |
| Neurological comorbidity * | 8 | 1.4 | 6 | -3 | 0.003 |
| Overweight patients (BMI: 24–30 Kg/m^2^) | 25.4 | 35.9 | 48.3 | -3.6 | < 0.001 |
| Absence of chronic hypertension | 24.3 | 31 | 43.5 | -3.7 | < 0.001 |
| Symptoms duration: 3^rd^ quartile (8.10) days | 16.7 | 9.9 | 20.2 | -3.9 | < 0.001 |
| Presence of CKD or PAD or complicated diabetes | 12.7 | 5.6 | 15.1 | -4.1 | < 0.001 |
| Compliance rs : 3^rd^ quartile (35.4, 44.9) mL/cm H_2_O | 17 | 12 | 24 | -4.3 | < 0.001 |
| Symptoms duration: 4^th^ quartile (10.27) days | 11 | 5.6 | 17.6 | -4.9 | < 0.001 |
| Charlson Comorbidity index ≥2 | 16.2 | 15.5 | 32.7 | -5.5 | < 0.001 |
| Female gender | 26.8 | 60.6 | 77.2 | -5.6 | < 0.001 |
| Symptoms duration: 1^st^ quartile (0.5) days | 13.7 | 11.3 | 28.1 | -5.7 | < 0.001 |
| Absence of diabetes | 26.6 | 63.4 | 81.2 | -6.5 | < 0.001 |
| Compliance rs: 4^th^ quartile (44.9.100) (mL/cm H_2_O) | 9.9 | 7.7 | 26.7 | -6.7 | < 0.001 |

* Stroke, dementia, or hemiplegia

Cla/Mod: Phenotype frequency in the variable modality
Mod/Cla: Variable modality frequency in the phenotype
Global: Variable modality overall frequency

v.test and p.value are obtained by performing a test corresponding to the hypergeometric distribution and calculating the probability of observing a more extreme value than that observed under the assumption of a draw with discount

Compliance rs: Compliance with the respiratory system

CKD: chronic kidney disease, BMI: Body mass index

PAD: Peripheral arterial disease.

ESM Table 3. Variables associated with phenotype 3

| **Phenotype 3 (N = 109)** | **Cla/Mod** | **Mod/Cla** | **Global** | **v.test** | **p.value** |
| --- | --- | --- | --- | --- | --- |
| Symptoms duration: 1^st^ quartile (0.5) days | 75.2 | 80.7 | 28.1 | 13.9 | < 0.001 |
| Charlson Comorbidity index ≥2 | 56.6 | 70.6 | 32.7 | 9.6 | < 0.001 |
| Presence of CKD or PAD or complicated diabetes | 74.6 | 43.1 | 15.1 | 8.8 | < 0.001 |
| Previous myocardial infarction or chronic cardiac failure | 65.3 | 29.4 | 11.8 | 6.1 | < 0.001 |
| PaO2/FiO2: <100 mmHg | 44.8 | 56 | 32.7 | 5.9 | < 0.001 |
| Chronic hypertension | 35.7 | 77.1 | 56.5 | 5.1 | < 0.001 |
| Neurological comorbidity * | 68 | 15.6 | 6 | 4.5 | < 0.001 |
| Compliance rs: 1^st^ quartile (12.9.28.7) (mL/cm H_2_O) | 41.9 | 40.4 | 25.2 | 4.1 | < 0.001 |
| Chronic pulmonary disease | 47.5 | 25.7 | 14.2 | 3.8 | < 0.001 |
| Age: 4^th^ quartile (71.90) years | 40.6 | 37.6 | 24.3 | 3.6 | < 0.001 |
| History of cancer | 45.6 | 19.3 | 11.1 | 3 | 0.003 |
| Absence of cancer | 23.8 | 80.7 | 88.9 | -3 | 0.002 |
| Charlson Comorbidity index =1 | 14.3 | 13.7 | 25.2 | -3.3 | < 0.001 |
| Age: 1^st^ quartile (38.55) | 13.3 | 12.8 | 25.2 | -3.6 | < 0.001 |
| Symptoms duration: 4^th^ quartile (10.27) days | 9.6 | 6.4 | 17.6 | -3.8 | < 0.001 |
| Absence of Chronic pulmonary disease | 22.7 | 74.3 | 85.8 | -3.8 | < 0.001 |
| Absence of neurological comorbidity* | 23.5 | 84.4 | 94 | -4.4 | < 0.001 |
| Symptoms duration: 3^rd^ quartile (8.10) days | 7.1 | 5.5 | 20.2 | -4.8 | < 0.001 |
| PaO2/FiO2: 100–150 mmHg | 13 | 19.3 | 38.7 | -5 | < 0.001 |
| Absence of chronic hypertension | 13.8 | 22.9 | 43.5 | -5.1 | < 0.001 |
| Absence of myocardial infarction and chronic cardiac failure | 21 | 70.6 | 88.2 | -6.1 | < 0.001 |
| Charlson Comorbidity index = 0 | 9.7 | 15.6 | 42.1 | -6.8 | < 0.001 |
| Symptoms duration: 2^nd^ quartile (5.8) days | 5.6 | 7.3 | 34.1 | -7.4 | < 0.001 |
| Absence of CKD, PAD, or complicated diabetes | 17.6 | 56.9 | 84.9 | -8.8 | < 0.001 |

* Stroke, dementia, or hemiplegia

Cla/Mod: Phenotype frequency in the variable modality
Mod/Cla: Variable modality frequency in the phenotype
Global: Variable modality overall frequency

v.test & p.value are obtained by performing a test corresponding to the hypergeometric distribution and calculating the probability of observing a more extreme value than that observed under the assumption of a draw with discount

Compliance rs: Compliance with the respiratory system

CKD: chronic kidney disease

PAD: Peripheral arterial disease.
